# Independent Prognostic Value of Natural Tooth Count Versus Posterior Teeth Occlusion in Resected Pancreatic Cancer

**DOI:** 10.1002/jhbp.70113

**Published:** 2026-04-23

**Authors:** Kaoru Seo, Kenichiro Uemura, Kenjiro Okada, Hiromi Nishi, Tatsuaki Sumiyoshi, Ryuta Shintakuya, Yoshiaki Murakami, Hiroyuki Kawaguchi, Shinya Takahashi

**Affiliations:** ^1^ Department of Surgery, Graduate School of Biomedical and Health Sciences Hiroshima University Hiroshima Japan; ^2^ Department of General Dentistry Hiroshima University Hospital Hiroshima Japan; ^3^ Digestive Disease Center Hiroshima Memorial Hospital Hiroshima Japan

**Keywords:** oral health, pancreatectomy, pancreatic cancer, prognosis, tooth loss

## Abstract

**Background/Purpose:**

Prognostic significance of preoperative patient‐related factors, including oral health status, in pancreatic ductal adenocarcinoma (PDAC) remains unclear. We evaluated the association between preoperative factors—particularly oral health parameters—and long‐term survival after curative PDAC resection.

**Methods:**

Patients who underwent radical pancreatectomy for PDAC (2014–2022) were retrospectively analyzed. Preoperative factors, including oral health status (natural tooth count and posterior teeth occlusion), clinical characteristics, and immuno‐nutritional markers, were assessed for their association with overall survival (OS). Multivariate models adjusted for established pathological and treatment‐related prognostic variables were further analyzed.

**Results:**

Of 343 eligible patients, 339 underwent preoperative dental examinations; 135 had ≤ 20 teeth and 204 had ≥ 21 teeth. Patients with ≤ 20 teeth had significantly poorer OS (median survival: 39.1 vs. 60.7 months, *p* < 0.001). Posterior teeth occlusion was not associated with OS. In multivariate analysis of preoperative factors, natural tooth count was independently associated with OS (*p* = 0.003). This association remained significant after adjustment for established prognostic variables (*p =* 0.016), whereas other immuno‐nutritional markers did not emerge as independent prognostic factors.

**Conclusions:**

Post‐PDAC resection, natural tooth count, but not posterior teeth occlusion, is independently associated with OS. Tooth count may reflect long‐term host vulnerability and serve as a prognostic marker in PDAC patients.

## Highlight

Seo and colleagues investigated the association of preoperative natural tooth count and posterior teeth occlusion with overall survival after pancreatic cancer resection. Having ≤20 teeth was independently associated with worse survival, whereas occlusion was not. Natural tooth count may reflect long‐term host vulnerability and serve as a simple prognostic indicator.

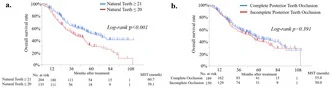

## 1 Introduction

Pancreatic ductal adenocarcinoma (PDAC) is among the most lethal forms of cancer, ranked as the fourth leading cause of cancer‐related deaths in the United States [1]. PDAC incidence is expected to increase worldwide; notably, it is expected to become the second leading cause of cancer mortality in the United States by 2030 [2]. Complete surgical resection is the only curative treatment for PDAC; however, only 20% of patients with PDAC are eligible for resection at the time of diagnosis [3]. Significant advancements in the field of adjuvant therapies for PDAC over the past decade have improved the prospects for curative treatment in patients with localized PDAC [4]. Despite these advancements, PDAC continues to have a poor 5‐year overall survival (OS) rate of approximately 10% [3].

Preoperative patient status, including immuno‐nutritional markers such as the modified Glasgow Prognostic Score (mGPS) and prognostic nutritional index (PNI) correlate with OS in patients with PDAC [5, 6]. Additionally, histologic and tumor‐related factors, including resectability, serum carbohydrate antigen (CA) 19–9 levels, lymph node involvement, surgical margin status, and adjuvant therapy, influence patient prognosis [4, 7, 8]. Given the increasing complexity and individualization of PDAC treatment, identifying patient‐specific prognostic factors among pretreatment clinical variables is critical for accurate risk stratification and optimal treatment planning.

Oral health has been increasingly recognized for its role in overall health and is associated with a variety of diseases [9]. Studies have shown the relationship between impaired oral health and gastrointestinal cancers [10]. For example, poor oral health is associated with unfavorable short‐term surgical outcomes following colorectal cancer treatment [11]. Furthermore, the number of natural teeth has been identified as an independent prognostic factor in esophageal cancer [12].

Previous studies on pancreatic cancer reported a higher tendency of adverse postoperative complications, including surgical site infections and post‐pancreatectomy hemorrhage, in patients with poor oral health [13, 14]. Several studies have reported periodontitis—a major cause of tooth loss—to be associated with increased PDAC risk [10, 15, 16]. These findings suggest a potential association between oral health and pancreatic cancer‐related outcomes, although the underlying mechanisms remain unclear.

However, the prognostic impact of oral health in patients undergoing curative PDAC resection remains largely unexplored. We hypothesized that preoperative oral health indicators, including number of natural teeth and posterior teeth occlusion, would be associated with the long‐term survival outcomes in this patient population. In this study, we aimed to evaluate these associations, with a particular focus on the prognostic significance of natural tooth count as a patient‐related background characteristic.

## 2 Methods

### 2.1 Study Design and Patient Selection

We retrospectively analyzed a cohort of patients diagnosed with pancreatic cancer who underwent radical pancreatectomy at the Department of Surgery in Hiroshima University Hospital between 2014 and 2022. Preoperative dental assessment of patients with PDAC has been a standard clinical practice in our department since December 2014. The prospectively collected preoperative oral health information was retrospectively analyzed. All eligible patients underwent a preoperative dental evaluation by a dentist. Four patients, who underwent emergency surgery and hence lacked preoperative dental records, were excluded from this analysis. Informed consent was waived owing to the anonymized nature of the data. This study was approved by the Institutional Review Board of Hiroshima University (approval number: E2024‐0226).

### 2.2 Preoperative Dental Evaluation and Tooth Count Classification

All patients underwent dental assessments and received oral care from dentists and dental hygienists within 1 week before surgery. Oral care included the removal of dental calculus, professional mechanical tooth cleaning, and tooth extraction for severe periodontitis. At the beginning of this process, the status of all 32 teeth was recorded in three categories: permanent teeth, missing teeth, and root fragments of permanent teeth. A natural tooth was considered present if categorized as a permanent tooth, and absent if categorized as either missing teeth or root fragments. In this study, third molars were excluded, resulting in a maximum possible tooth count of 28 per patient. Patients were classified into two groups: the ≤ 20 teeth group (20 or fewer teeth) and the ≥ 21 teeth group (21 or more teeth). A cut‐off value of 21 teeth was adopted based on previous studies which reported that the presence of ≥ 21 teeth represents a functional dentition and is associated with better general health [9, 17, 18].

Additionally, the status of posterior teeth occlusion was evaluated because mastication is a fundamental function of teeth, and molar and premolar teeth play a central role in this function. This assessment was performed using the total number of posterior teeth, including both natural and prosthetically replaced teeth. Patients with all posterior teeth present, either natural or prosthetically restored, were classified as having complete posterior teeth occlusion. In contrast, patients missing one or more posterior teeth, regardless of prosthetic replacement, were classified as having incomplete posterior teeth occlusion.

Finally, we combined the total number of natural teeth and the status of posterior teeth occlusion to classify patients into four groups: Group A comprised patients with ≥ 21 natural teeth with complete posterior teeth occlusion; Group B comprised patients with ≥ 21 natural teeth with incomplete posterior teeth occlusion; Group C comprised patients with ≤ 20 natural teeth with complete posterior teeth occlusion; and Group D comprised patients with ≤ 20 natural teeth with incomplete posterior teeth occlusion. Subsequently, OS was compared among these four groups.

### 2.3 Definitions

Sex was defined as that assigned at birth and age as patients' age at the time of surgery. Tumor stage, lymph node involvement, and overall cancer staging were determined using the 8th edition of the Union for International Cancer Control (UICC) classification system [19]. Surgical complications were categorized using the Clavien–Dindo classification system [20]. Resectability status was determined based on criteria outlined in the National Comprehensive Cancer Network guidelines [21]. Patient nutritional and inflammatory markers were assessed for their prognostic value. The mGPS and Controlling Nutritional Status (CONUT) scores were calculated as previously described [5, 22]. Onodera's PNI was assessed as an indicator of nutritional status, with lower values indicating malnutrition [6]. The prognostic relevance of the neutrophil‐to‐lymphocyte ratio (NLR) and C‐reactive protein‐to‐albumin ratio (CAR) was also examined [23, 24]. A history of cardiovascular disease included past incidence of angina pectoris, myocardial infarction, arrhythmia, or a major vascular disease. Additionally, pulmonary conditions comprised chronic obstructive pulmonary disease, asthma, interstitial pneumonia, and lung cancer. Renal dysfunction was defined as an estimated glomerular filtration rate of < 60 mL/min/1.73 m^2^.

### 2.4 Data Collection

Data comprising patient demographics, clinical characteristics, and laboratory findings were collected. Patient data including age, sex, body mass index (BMI), and American Society of Anesthesiologists physical status classification (ASA‐PS) were recorded. Laboratory results, including preoperative serum CA19‐9 levels, serum albumin concentrations, and other biochemical markers, were obtained. Surgical variables, such as operative procedures, complications, resectability status, and pathological findings (lymph node status, resection margins, and UICC pT stage), were documented. Data pertaining to neoadjuvant chemotherapy (NAC) and/or postoperative adjuvant chemotherapy were collected. OS was measured from the date of pancreatectomy until either death or the latest follow‐up. BMI and ASA‐PS were recorded 1–2 weeks before surgery, whereas all other laboratory data were collected within 1 week before surgery.

### 2.5 Preoperative Chemotherapy and Criteria for Surgery

Gemcitabine‐based NAC was administered to patients with resectable (R) PDAC since 2020 and to those with borderline resectable (BR) PDAC since 2009. Patients with R or BR PDAC underwent NAC followed by radiographic assessment. Curative‐intent pancreatectomy was performed when institutional criteria were met, and complete resection was considered feasible. Patients with unresectable PDAC received systemic chemotherapy. Conversion surgery was considered when unresectable factors, including distant metastasis, resolved following treatment and curative resection was deemed feasible. Adjuvant chemotherapy was administered after curative‐intent pancreatectomy.

### 2.6 Statistical Analysis

Data analyses and visualizations were conducted using JMP18 software (SAS Institute, Cary, NY, USA). Categorical variables are presented as counts with corresponding percentages, whereas continuous variables as medians and interquartile ranges (IQRs). Differences in patient characteristics between subgroups were assessed using the Mann–Whitney U test. Survival analyses were conducted using the Kaplan–Meier method, with comparisons made using the log‐rank test. To assess the prognostic significance of relevant variables, univariable and multivariable Cox proportional hazards regression models were used. Significant variables from the univariable analysis were included in the multivariable analysis. An additional multivariable model was constructed by incorporating established pathological and treatment‐related factors together with preoperative prognostic factors independently associated with survival to assess whether these associations were maintained after adjustment. The association between natural tooth count and patient characteristics was evaluated using a multivariable logistic regression model, with detailed results presented in the Table S1. Hazard ratios (HRs) are presented along with 95% confidence intervals (CIs). A two‐sided *p*‐value of < 0.05 was considered statistically significant.

## 3 Results

### 3.1 Participants

**FIGURE 1:**
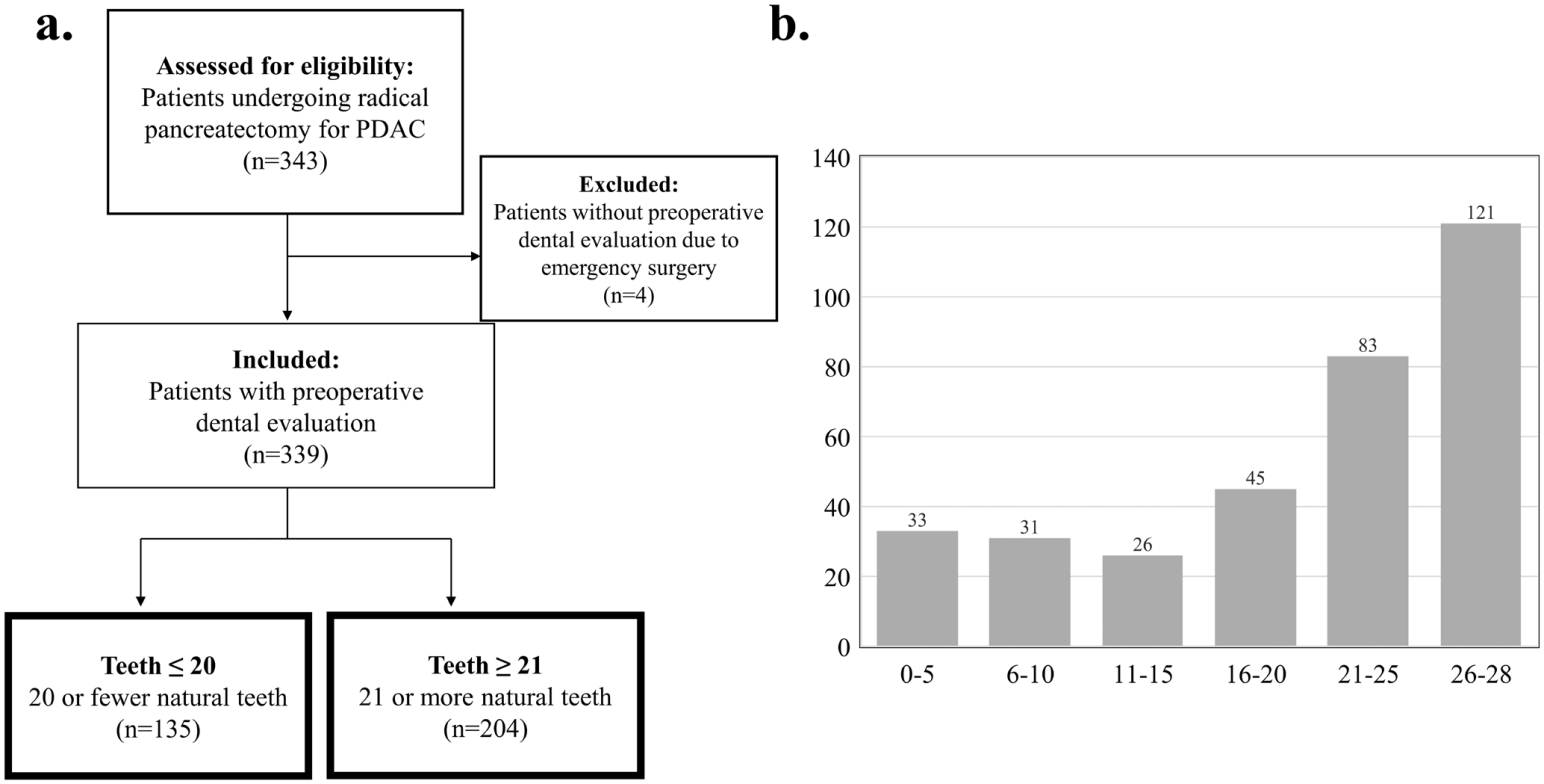
Study flowchart and distribution of natural teeth. (a) Flow diagram of patient selection. (b) Distribution of the number of natural teeth in patients included in the study.

A total of 343 consecutive patients with PDAC underwent radical pancreatectomy in our department between 2014 and 2022. After excluding 4 patients who lacked preoperative dental evaluation data owing to emergency surgery, 339 were included in the final analysis. Among these, 135 patients were classified into the ≤ 20 teeth group and 204 into the ≥ 21 teeth group (Figure 1a). The distribution pattern of participants' tooth count is shown in Figure 1b. Table 1 summarizes the clinicopathological features of the patient cohort (median age, 71.0 years [IQR: 63.0–77.0 years]; females, 159 patients [46.9%]; median number of natural teeth, 23.0 [IQR: 15.0–27.0]; and median preoperative serum CA19‐9 level, 38.0 U/mL [IQR: 8.0–180.5 U/mL]). The resectability status was classified as R in 225 patients (66.4%) and BR or unresectable‐locally advanced (URLA) in 114 patients (33.6%). Surgery types included pancreatoduodenectomy (*n* = 229, 67.6%), distal pancreatectomy (*n* = 108, 31.8%), and total pancreatectomy (*n* = 2, 0.6%). NAC was administered to 161 patients, representing 47.5% of all cases. A total of 245 (72.3%) patients completed adjuvant chemotherapy.

**TABLE 1:** Baseline Characteristics of Included Patients.

Variables	Total (*N* = 339)
Age (years), median	71.0 (IQR, 63.0–77.0)
Sex, female	159 (46.9)
BMI (kg/*m* ^2^), median	21.3 (IQR, 19.1–23.7)
ASA‐PS, ≥ 3	15 (4.4)
Diabetes mellitus	130 (38.3)
Cardiovascular disease	30 (8.9)
Pulmonary disease	25 (7.4)
Renal dysfunction	71 (20.9)
Preoperative CA19‐9 level (U/mL), median	38.0 (IQR, 8.0–180.5)
Resectability status	
R	225 (66.4)
BR, URLA	114 (33.6)
Tumor location	
Head	224 (66.1)
Body, tail	115 (33.9)
Neoadjuvant chemotherapy	161 (47.5)
Number of natural teeth, median	23.0 (IQR, 15.0–27.0)
≤ 20	135 (39.8)
≥ 21	204 (60.2)
Posterior teeth occlusion	
Complete	189 (55.8)
Incomplete	150 (44.2)
Nutritional status	
mGPS, 0	259 (76.4)
PNI, median	39.0 (IQR, 36.0–42.0)
NLR, median	2.3 (IQR, 1.7–3.2)
CONUT score, ≤ 4	307 (90.6)
CAR, median	0.03 (IQR, 0.01–0.10)
Serum albumin level (g/dL), median	3.9 (IQR, 3.6–4.2)
Surgical procedures	
PD	229 (67.6)
DP	108 (31.8)
TP	2 (0.6)
pT [UICC]	
T1, T2	270 (79.6)
T3, T4	69 (20.4)
Lymph node metastasis	
Positive	220 (64.9)
Negative	119 (35.1)
pStage [UICC]	
I, II	251 (74.0)
III, IV	88 (26.0)
Residual tumor status, R0	273 (80.5)
Tumor differentiation, well differentiated	84 (24.8)
Postoperative complication, ≥ CD grade 3	55 (16.2)
Adjuvant chemotherapy, complete	245 (72.3)

*Note:* Data are presented as *n* (%) or median (interquartile range).

Abbreviations: ASA‐PS, American Society of Anesthesiologists physical status classification system; BMI, body mass index; BR, borderline resectable; CA19‐9, carbohydrate antigen 19–9; CAR, C‐reactive protein‐to‐albumin ratio; CD, Clavien–Dindo; CONUT, Controlling Nutritional Status; DP, distal pancreatectomy; mGPS, modified Glasgow Prognostic Score; NLR, neutrophil‐to‐lymphocyte ratio; PD, pancreatoduodenectomy; PNI, Prognostic Nutritional Index; R, resectable; TP, total pancreatectomy; UICC, Union for International Cancer Control; URLA, unresectable‐locally advanced.

### 3.2 Survival Analysis

**FIGURE 2:**
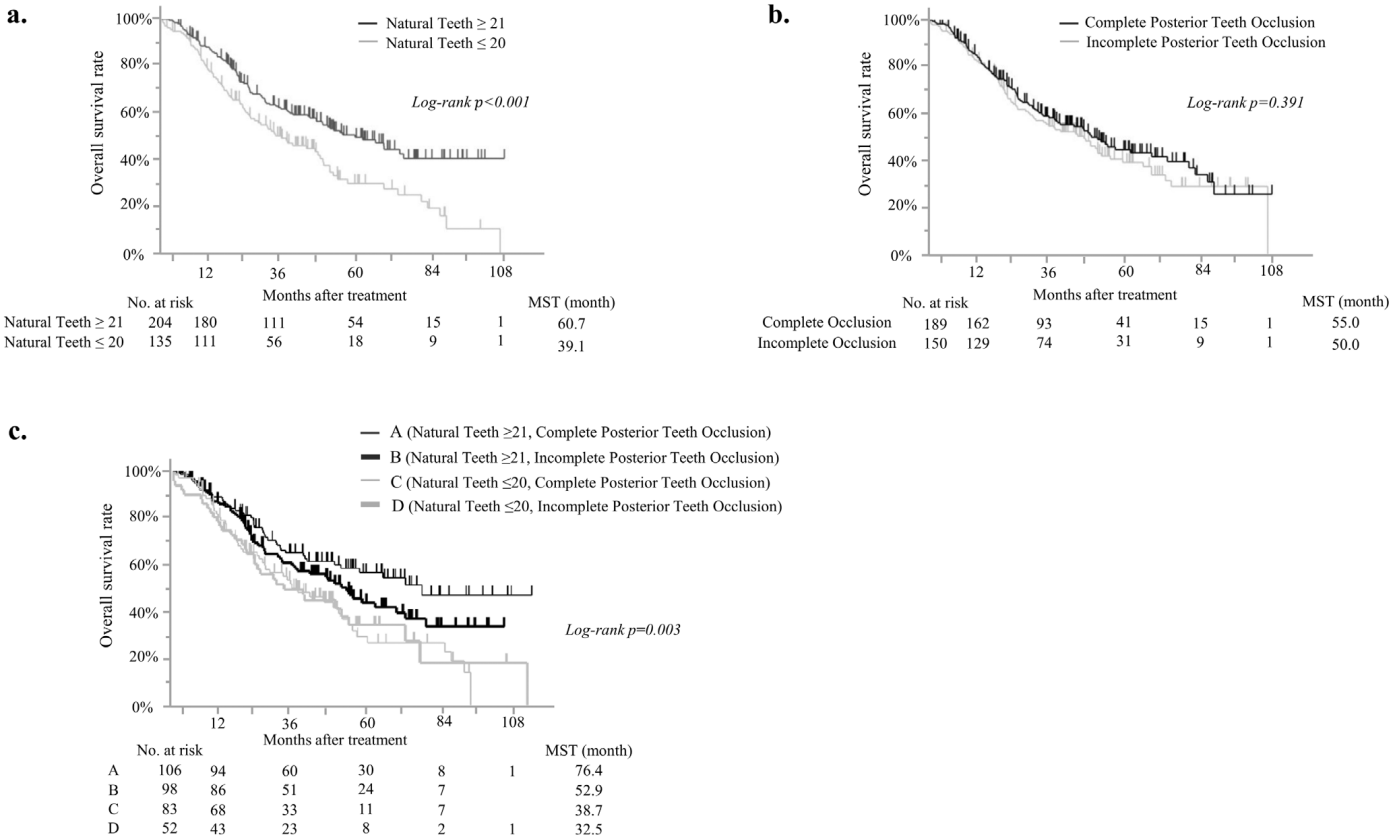
Kaplan–Meier survival curves. (a) Median overall survival is 60.7 months in the ≥ 21 teeth group and 39.1 months in the ≤ 20 teeth group (*p* < 0.001). The hazard ratio for mortality in the ≤ 20 teeth group compared with the ≥ 21 teeth group is 1.67 (95% CI 1.24–2.25; *p* < 0.001). (b) Median overall survival is 55.0 months in the complete posterior teeth occlusion group and 50.0 months in the incomplete posterior teeth occlusion group (*p* = 0.391). The hazard ratio for mortality in the complete posterior teeth occlusion group compared with the incomplete posterior teeth occlusion group is 0.88 (95% CI 0.65–1.18; *p* = 0.392). (c) Median overall survival is 76.4 months, 52.9 months, 38.7 months, and 32.5 months in Groups A, B, C, and D, respectively (*p* = 0.003). The hazard ratio for mortality in Group A compared with Groups B, C, and D is 0.60 (95% CI 0.42–0.84; *p* = 0.003).

The median follow‐up duration was 35.5 months (IQR: 19.2–56.7 months). Patients in the ≥ 21 teeth group exhibited a significantly longer median survival time (MST) of 60.7 months than those in the ≤ 20 teeth group (39.1 months; *p* < 0.001, Figure 2a). In contrast, no difference in OS was observed between patients with complete and incomplete posterior teeth occlusion (55.0 vs. 50.0 months, *p* = 0.391, Figure 2b). When comparing Groups A–D (classified by natural tooth count and posterior teeth occlusion), OS differed significantly among the groups; Group A exhibited the most favorable prognosis, whereas Group D revealed the poorest (MST: 76.4 vs. 32.5 months, *p* = 0.003, Figure 2c).

Univariable analysis identified several preoperative factors to be significantly associated with OS (Table 2). Multivariable analysis revealed that having ≤ 20 teeth was independently associated with poorer OS (HR: 1.57; 95% CI: 1.16–2.12; *p* = 0.003). Other independent poor prognostic factors included ASA‐PS ≥ 3, preoperative CA19‐9 level ≥ 37 U/mL, BR or URLA resectability status, and tumor location in the head of pancreas. Number of natural tooth count remained significantly associated with OS after adjustment for preoperative prognostic factors, pathological factors, and treatment‐related factors (HR: 1.47; 95% CI: 1.07–2.01; *p* = 0.016) (Table 3).

**TABLE 2:** Univariate and Multivariate Analyses of Preoperative Prognostic Factors for Overall Survival.

Variables	Univariate			Multivariate		
	Entire (*n* = 339)	MST (month)	*p*	HR	95% CI	*p*
Age (years)			0.085			
≤ 70	157 (46.3)	55.2				
> 70	182 (53.7)	49.7				
Sex			0.398			
Male	180 (46.9)	55.0				
Female	159 (53.1)	50.7				
BMI (kg/m^2^)			0.079			
< 22	189 (55.8)	41.3				
≥ 22	150 (44.2)	56.7				
ASA‐PS			0.022			0.047
≤ 2	324 (95.6)	52.9				
≥ 3	15 (4.4)	23.0		1.88	1.01–3.52	
Diabetes mellitus			0.153			
Yes	130 (38.3)	46.8				
No	209 (61.7)	55.0				
Cardiovascular disease			0.657			
Yes	30 (8.9)	32.0				
No	309 (91.1)	52.7				
Pulmonary disease			0.166			
Yes	25 (7.4)	23.7				
No	314 (92.6)	52.7				
Renal dysfunction			0.734			
Yes	71 (20.9)	56.7				
No	268 (79.1)	51.3				
Preoperative CA19‐9 level (U/mL)			0.004			0.008
< 37	165 (48.7)	59.0				
≥ 37	174 (51.3)	38.1		1.51	0.48–0.90	
Resectability			0.002			< 0.001
R	225 (66.4)	60.7				
BR, URLA	114 (33.6)	32.5		1.78	1.30–2.43	
Tumor location			0.010			0.019
Head	224 (66.1)	47.3		1.50	1.07–2.09	
Body, tail	115 (33.9)	76.4				
Neoadjuvant chemotherapy			0.443			
Yes	161 (47.5)	47.9				
No	178 (52.5)	55.2				
Number of natural teeth			< 0.001			0.003
≤ 20	135 (39.8)	39.1		1.57	1.16–2.12	
≥ 21	204 (60.1)	60.7				
Posterior teeth occlusion			0.391			
Complete	189 (55.8)	55.0				
Incomplete	150 (44.2)	50.0				
mGPS			0.004			0.121
0	259 (76.4)	56.3				
1,2	80 (23.6)	32.0		1.68	0.87–3.22	
PNI			0.014			0.309
< 45	302 (89.1)	50.7		1.35	0.41–1.32	
≥ 45	37 (10.9)	74.2				
NLR			0.318			
≤ 4	284 (83.8)	52.9				
> 4	55 (16.2)	37.4				
CONUT			0.031			0.239
≤ 4	307 (90.6)	52.9				
> 4	32 (9.4)	29.2		1.45	0.78–2.67	
CAR			0.085			
≤ 0.03	155 (45.7)	55.7				
> 0.03	184 (54.3)	50.0				
Serum albumin level (g/dL)			0.013			0.436
< 3.5	66 (19.5)	32.4		0.73	0.63–2.95	
≥ 3.5	273 (80.5)	54.3				

*Note:* Data are presented as *n* (%).

Abbreviations: ASA‐PS, American Society of Anesthesiologists physical status classification system; BMI, body mass index; BR, borderline resectable; CA19‐9, carbohydrate antigen 19–9; CAR, C‐reactive protein‐to‐albumin ratio; CI, confidence interval; CONUT, Controlling Nutritional Status; HR, hazard ratio; mGPS, modified Glasgow Prognostic Score; MST, median survival time; NLR, neutrophil‐to‐lymphocyte ratio; PNI, Prognostic Nutritional Index; R, resectable; URLA, unresectable‐locally advanced.

**TABLE 3:** Multivariate Analyses of Overall Survival Including Preoperative Prognostic Factors and Established Pathological and Treatment‐Related Variables.

Variables	HR	95% CI	*p*‐value
ASA‐PS, ≥ 3	1.34	0.71–2.51	0.365
Natural Teeth, ≤ 20	1.47	1.07–2.01	0.016
Preoperative CA19‐9 level, ≥ 37 U/mL	1.16	0.84–1.59	0.362
Resectability, R	0.79	0.57–1.09	0.154
Tumor location, head	1.42	1.00–2.01	0.048
pT [UICC], T1 T2	0.68	1.00–2.12	0.048
Lymph node metastasis, positive	2.37	1.63–3.46	< 0.001
Residual tumor status, R0	0.55	0.39–0.79	0.001
Tumor differentiation, well differentiated	0.48	0.31–0.75	0.001
Adjuvant chemotherapy, complete	0.21	0.15–0.29	< 0.001

Abbreviations: 95% CI, 95% confidence interval; ASA‐PS, American Society of Anesthesiologist–Physical Status Classification System; CA19‐9, carbohydrate antigen 19–9; HR, hazard ratio; R, resectable; UICC, Union for International Cancer Control.

## 4 Discussion

Our findings indicate that among patients with PDAC, natural tooth count, but not posterior teeth occlusal status, is independently associated with poorer OS following radical PDAC resection. This association between natural tooth count and OS was preserved even after adjustment for established pathological and treatment‐related variables.

Previous studies on the relationship between oral health and PDAC have primarily focused on either the risk of PDAC development or short‐term postoperative outcomes [10, 13, 14]. To the best of our knowledge, no previous study has evaluated the prognostic significance of the number of natural teeth in patients undergoing curative resection for PDAC.

Tooth loss is closely linked to systemic conditions such as diabetes and cardiovascular disease [18]. Epidemiological studies have reported that individuals with more than 21 natural teeth are more likely to exhibit better overall health status [9]. In addition, functional studies have suggested that at least 21 natural teeth are required to maintain adequate mastication efficiency without relying on dentures or other prosthetic devices [17]. Based on this evidence, we defined 21 teeth as the clinically meaningful threshold and set the cut‐off value at 21 teeth. Moreover, posterior teeth occlusion, constructed from both natural and prosthetically restored teeth, plays a central role in masticatory function; therefore, the association between the posterior teeth occlusal status and survival outcomes was also compared [25, 26].

Patients with a higher number of natural teeth (≥ 21) demonstrated significantly better OS than did those with fewer teeth (≤ 20), with MSTs of 60.7 and 39.1 months, respectively. Furthermore, patients with fewer natural teeth tended to be older and had more comorbidities, including diabetes mellitus and renal dysfunction (Supporting Table 1). They also exhibited poorer immuno‐nutritional profiles, reflected by lower PNI and higher mGPS, NLR, CONUT, and CAR scores (Supporting Table 1). This observation is clinically relevant, because previous studies have demonstrated that these markers are closely associated with PDAC prognosis [5, 6, 22, 23, 24]. Moreover, patients with fewer natural teeth had a lower completion rate of adjuvant chemotherapy, consistent with earlier reports indicating that patients with compromised immuno‐nutritional status are less likely to complete adjuvant therapy [27, 28]. Despite these associations, most of the immuno‐nutritional markers did not emerge as independent prognostic factors in this cohort. Although ASA‐PS was significantly associated with OS among preoperative factors, after adjustment for established pathological and treatment‐related variables, the number of natural teeth was the only non‐tumor‐related variable independently associated with OS. This apparent discrepancy between the prognostic impact of natural tooth count and that of immuno‐nutritional indices may reflect differences in temporal scope: while immuno‐nutritional indices capture short‐term physiological status at a single time point, tooth count represents cumulative systemic vulnerability accrued over decades. Natural tooth count is a cumulative and largely irreversible marker influenced by a broad range of factors, including long‐term nutritional status, chronic inflammatory burden, frailty, health‐related behaviors such as smoking, socioeconomic status, and access to medical and dental care [9, 18, 25]. These life‐course determinants of health are difficult to quantify individually in retrospective surgical cohorts. In this context, natural tooth count should be regarded as a host‐related background characteristic that integrates the cumulative effects of these unmeasured or partially measured systemic conditions. Thus, the observed association between tooth count and survival is likely reflecting differences in long‐standing patient vulnerability rather than a direct biological effect of tooth loss itself.

In contrast to natural tooth count, posterior teeth occlusion was not associated with OS in this cohort. This finding appears inconsistent with those of previous reports linking occlusal teeth function to nutritional status and mortality; however, several considerations may explain this discrepancy [25, 26, 29]. Posterior teeth occlusion represents a functional and potentially reversible condition that can be modified through dental intervention, including prosthetic rehabilitation, particularly in the perioperative period. Therefore, a single preoperative assessment of occlusal status may not adequately capture longitudinal status. Importantly, masticatory function or occlusal force were not directly assessed in the present study. Accordingly, natural tooth count was positioned as an indirect surrogate marker reflecting oral function capacity rather than as a direct measure of masticatory performance. This distinction is particularly relevant when interpreting the prognostic implications of oral health measures in relation to long‐term outcomes. Moreover, posterior teeth occlusion can be modified in the relatively short term, whereas tooth loss is largely irreversible and accumulates over decades. From this perspective, posterior teeth occlusion may be less suitable as a marker of long‐standing host status for long‐term oncologic outcomes. Therefore, the absence of an association between posterior teeth occlusion and survival in the present study does not contradict the potential importance of oral function in general. Instead, it underscores the importance of distinguishing between transient functional status and stable background characteristics when evaluating prognostic markers in patients with PDAC. When stratified by natural tooth count and posterior teeth occlusal status, patients in Group A (≥ 21 natural teeth with complete posterior teeth occlusion) had more than twice the OS of those in Group D (≤ 20 natural teeth with incomplete posterior teeth occlusion). This survival advantage may reflect a more favorable oral environment and a more stable masticatory function, which are generally preserved in individuals with a higher natural tooth count. Taken together, these findings suggest that tooth loss, as a marker of long‐term oral health status, may be more strongly associated with survival than posterior teeth occlusal status assessed at a single time point. However, further studies are needed to clarify the mechanisms underlying these associations and to determine whether improvements in oral health can contribute to better outcomes.

Beyond the scope of the present analysis, previous studies have shown a relationship between poor oral health and PDAC. Epidemiological studies have reported that patients with periodontitis, a major cause of tooth loss, have an increased risk of developing PDAC [10, 30]. In addition, several experimental studies have demonstrated that periodontal pathogens such as *P. gingivalis* can reach pancreatic tissue and influence pancreatic carcinogenesis by promoting the progression from pancreatic intraepithelial neoplasia to PDAC [15, 16]. Although these findings do not establish causality in humans, they provide a biologically plausible link between chronic oral inflammation and occurrence of PDAC. Importantly, we did not investigate the causes of tooth loss or the presence of periodontal infection in the present study. Therefore, natural tooth count was not positioned as a direct biological measure of infection nor inflammation. Further translational and clinical studies are required to clarify the mechanisms linking oral health status with PDAC outcomes.

This study has some limitations. First, this was a single‐center, retrospective cohort study comprising patients of Japanese ethnicity, limiting the generalizability of the findings. Furthermore, although multivariable analyses adjusted for established pathological and treatment‐related prognostic variables were performed, residual confounding by unmeasured factors could not be excluded. In particular, detailed smoking history was not available in this cohort, which may have influenced both oral health status and pancreatic cancer outcomes. Other unmeasured factors, including socioeconomic status, cumulative comorbidity burden, and access to medical and dental care, may also have contributed to the survival. Therefore, future multicenter studies with international cohorts are warranted to validate our findings. Second, nearly all patients received preoperative oral assessments and care; thus, comparison with a group without access to oral care was not feasible, and the influence of oral management on postoperative outcomes could not be determined. Third, the potential effects of NAC on oral health were not evaluated. Although dental assessment was routinely performed before NAC at our institution, changes in oral condition during therapy may not have been fully captured. Fourth, denture‐related factors—including type, fit, and frequency of use—were not assessed despite their potential influence on nutritional intake [25]. Finally, the specific causes of tooth loss were not examined. Tooth loss is multifactorial, and the contribution of oral diseases such as periodontitis and dental caries to tooth loss in this cohort was not assessed [9]. Future multicenter prospective studies are warranted to address these limitations and further clarify the relationship between oral health and PDAC outcomes.

Despite these limitations, the present study has important clinical implications. Our findings suggest that preoperative natural tooth count is independently associated with survival and may serve as a simple, objective, and readily available patient‐related background marker for risk stratification in patients undergoing curative resection for PDAC. Tooth loss reflects long‐standing host vulnerability that is not fully captured by conventional clinicopathological variables and may integrate cumulative lifetime exposures, including oral health, nutritional reserve, and lifestyle factors. Importantly, natural tooth count should not be interpreted as a modifiable therapeutic target or as evidence of a causal relationship with survival, but rather as a prognostic indicator that may help identify patients at higher risk of poor postoperative outcomes. Incorporating oral health assessment into the preoperative evaluation may therefore provide complementary prognostic information. Further multicenter prospective studies are needed to validate these findings, explore underlying mechanisms, and determine whether comprehensive oral health optimization can contribute to improved outcomes in patients with PDAC.

## Author Contributions

Conceptualization: K.S., K.U., K.O., and H.N. Data curation: K.S., K.O., H.N., T.S., and R.S. Formal analysis: K.S., K.U., K.O., H.N., R.S., and Y.M. Investigation: K.U., K.O., H.N., T.S., Y.M., and H.K. Methodology: K.U., K.O., T.S., and Y.M. Project administration: K.S., K.O., and H.N. Resource: K.U., H.N., Y.M., and H.K. Supervision: K.U., K.O., H.N., Y.M., and H.K. Validation: T.S., R.S. Visualization: K.S., K.O., and R.S. Writing – original draft: K.S., K.U., K.O., and H.N.

## Funding

The authors have nothing to report.

## Ethics Statement

The study was approved by the Institutional Review Board of Hiroshima University (approval number: E2024‐0226).

## Consent

The requirement for informed consent was waived owing to the anonymized nature of the data.

## Conflicts of Interest

The authors declare no conflicts of interest.

## Supporting information


**Table S1** Univariate and Multivariate Analyses of Factors Associated with 20 or Fewer Natural Teeth (Teeth ≤ 20).

## Data Availability

The data that support the findings of this study are available on request from the corresponding author. The data are not publicly available due to privacy or ethical restrictions.
