# Supplementary material for: Independent Prognostic Value of Natural Tooth Count Versus Posterior Teeth Occlusion in Resected Pancreatic Cancer
Source: J Hepatobiliary Pancreat Sci. 2026 Apr 23;33(9):720–9. doi: 10.1002/jhbp.70113 (PMC13595496; doi:10.1002/jhbp.70113)
Supplement: Supplementary file 1 [file JHBP-33-720-s001.docx]

**Supporting Table 1. Univariate and Multivariate Analyses of Factors Associated with 20 or Fewer Natural Teeth (Teeth≤20)**

| Variables | Univariate | | | Multivariate | |
| --- | --- | --- | --- | --- | --- |
|  | Teeth≤20  n=135 | Teeth≥21  n=204 | *p*-value | OR (95%CI) | *p*-value |
| Age, >70 years | 92 (68%) | 90 (44%) | **<0.001** | 2.05 (1.24–3.39) | **0.005** |
| Sex, male | 78 (58%) | 102 (50%) | 0.160 |  |  |
| BMI, ≥22kg/m^2^ | 60 (44%) | 90 (44%) | 0.953 |  |  |
| ASA-PS, ≥3 | 4 (3%) | 11 (5%) | 0.275 |  |  |
| Diabetes mellitus | 61 (45%) | 69 (34%) | **0.036** | 1.35 (0.84–2.18) | 0.213 |
| Cardiovascular disease | 17 (13%) | 13 (6%) | 0.051 |  |  |
| Pulmonary disease | 11 (8%) | 14 (7%) | 0.659 |  |  |
| Renal dysfunction | 36 (27%) | 35 (17%) | **0.037** | 1.28 (0.72–2.29) | 0.395 |
| Preoperative CA19-9, ≥37U/mL | 74 (55%) | 100 (49%) | 0.296 |  |  |
| Resectability, R | 86 (64%) | 139 (68%) | 0.399 |  |  |
| Tumour location, head | 88 (65%) | 136 (67%) | 0.778 |  |  |
| Tumour size, >20mm | 99 (73%) | 145 (71%) | 0.138 |  |  |
| Neoadjuvant chemotherapy | 61 (45%) | 100 (49%) | 0.489 |  |  |
| mGPS, 1 or 2 | 41 (30%) | 39 (19%) | **0.018** | 1.22 (0.38–3.92) | 0.743 |
| PNI, <45 | 126 (93%) | 176 (86%) | **0.036** | 1.61 (0.68–3.77) | 0.277 |
| NLR, >4 | 32 (24%) | 23 (11%) | **0.003** | 2.03 (1.09–3.78) | **0.025** |
| CONUT, >4 | 18 (13%) | 14 (7%) | **0.049** | 1.18 (0.46–3.06) | 0.728 |
| CAR, >0.03 | 84 (62%) | 100 (49%) | **0.017** | 1.47 (0.90–2.40) | 0.125 |
| Serum albumin level, <3.5g/dL | 35 (26%) | 31 (15%) | **0.016** | 1.02 (0.27–3.78) | 0.980 |
| Adjuvant chemotherapy, complete | 89 (66%) | 156 (76%) | **0.035** | 0.78 (0.47–1.30) | 0.340 |
| Postoperative complication,  ≥CD grade 3 | 24 (18%) | 31 (15%) | 0.530 |  |  |

OR indicates odds ratio; 95% CI, 95% confidence interval; BMI, body mass index; ASA-PS, American Society of Anesthesiologist-Physical status classification system; CA19-9, carbohydrate antigen 19-9; R, resectable; mGPS, modified Glasgow Prognostic Score; PNI, Prognostic Nutritional Index; NLR, neutrophil-to-lymphocyte ratio; CONUT, Controlling Nutritional Status; CAR, C-reactive-protein-to albumin ratio; CD, Clavien–Dindo
